# Complete Genome Sequences of Two Strains of “*Candidatus* Filomicrobium marinum,” a Methanesulfonate-Degrading Species

**DOI:** 10.1128/genomeA.00160-15

**Published:** 2015-05-07

**Authors:** Ana C. Henriques, Paolo De Marco

**Affiliations:** Centro de Investigação em Ciências da Saúde (CICS), Instituto Superior de Ciências da Saúde-Norte, CESPU, Rua Central de Gandra, Paredes, Portugal

## Abstract

Two novel methanesulfonate-degrading bacterial strains of “*Candidatus* Filomicrobium marinum” (strains Y and W) were isolated from a marine water enrichment, and their complete genome sequences are presented here. These are the first full genomes reported for the genus *Filomicrobium* and for methanesulfonate (MSA)-degrading bacteria.

## GENOME ANNOUNCEMENT

Methanesulfonate (MSA) is quantitatively a very relevant compound in the biogeochemical sulfur cycle (1–6). MSA can be used aerobically as a sulfur source by some bacteria (6), while several methylotrophic species isolated from different environments can grow using MSA as the sole source of carbon and energy (7–15). The strains analyzed at the molecular level contain an inducible MSA monooxygenase that oxidizes MSA to the central methylotrophic intermediate formaldehyde (7–13).

The complete genomic sequences of two such novel marine methylotrophs are described here. They were isolated from an enrichment from North Atlantic surface seawater with MSA as sole source of C (16); one produced white colonies (“*Candidatus* Filomicrobium marinum” strain W), and one produced yellow colonies (“*Candidatus* Filomicrobium marinum” strain Y). The genomes were sequenced by Molecular Research LP (Shallowater, TX, USA) using the MiSeq Illumina sequencing platform. The coverages were 294× and 381×, respectively. Sequence reads were assembled using the NGen assembler (DNAStar, Inc.). The numbers of assembly contigs generated were 2 for strain Y and 4 for strain W. Gaps were closed by PCR and Sanger sequencing. The two sequences were reconstructed into circular genomes of 3,969,942 bp (strain Y) and 3,969,936 bp (strain W). Both genomes were analyzed and annotated using the MicroScope platform (https://www.genoscope.cns.fr/agc/microscope/home/index.php) (17).

The two 16S rRNA genes are identical to each other, and BLASTn (http://www.ncbi.nlm.nih.gov/BLAST/) (18) and Ribosomal Database Project (RDP) (http://rdp.cme.msu.edu/) (19) analyses show a very clear association with other *Filomicrobium* organisms (highest identity with *Filomicrobium insigne* strain SLG5B-19, 97.9%). These are the first genomes reported for the *Filomicrobium* genus and for MSA-degrading bacteria.

The 2 genomes are almost identical, differing in only 29 single-nucleotide polymorphisms (SNPs) (of which 5 are ambiguously scored bases) and a large 1,205,437-bp inversion (20); for convenience, we will describe strain Y only. The genome included 4,112 genomic objects (4,045 coding sequences [CDSs], 8 fragments of CDS, 8 genes for miscellaneous RNA, 3 genes for rRNA [one 16S, one 23S, and one 5S], and 48 tRNAs). The G+C genomic content is 57.29%. Of the CDSs, 2,886 (71%) are categorized in at least one COG group.

Genes involved in most major metabolic pathways were found, such as those for the tricarboxylic acid (TCA) cycle, pentose phosphate pathway, amino acid metabolism, and biosynthesis. Genes coding for an electron transport chain and oxidative phosphorylation are also present, as well as those encoding dimethyl sulfoxide (DMSO) reductase and nitrate reductase. However, the glycolytic pathways are not complete, and no pathways for CO_2_ fixation were found.

As expected, methylotrophic genes are present: methanol dehydrogenase encoded by the *mxa* and *xox* genes (21), methylamine dehydrogenase *mau* genes (22), a MSA monooxygenase operon (*msmABCD*) (7), and a MSA transport operon (*msmEFGH*) (23). Two open reading frames (ORFs) were found downstream of *msmH* encoding a SoxDC sulfite dehydrogenase (24).

Genes involved in the serine cycle are present. Isocitrate lyase was not found, but most of the enzymes required for the ethylmalonyl-coenzyme A (CoA) pathway (25, 26) were predicted. Regarding formaldehyde dissimilation, both the tetrahydrofolate and tetrahydromethanopterin pathways were found. Genes encoding formate dehydrogenase are also present.

These features bring new insights into the genomic potential of members of the genus *Filomicrobium* and MSA-degrading bacteria in general.

### Nucleotide sequence accession numbers. 

The complete genome sequences of strains Y and W were deposited in the European Nucleotide Archive under BioProject number PRJEB8348 and accession numbers LN829119 and LN829118, respectively.
